# NCC Based Correspondence Problem for First- and Second-Order Graph Matching †

**DOI:** 10.3390/s20185117

**Published:** 2020-09-08

**Authors:** Beibei Cui, Jean-Charles Créput

**Affiliations:** 1 College of Electrical Engineering, Henan University of Technology, Zhengzhou 450001, Henan, China; 2 CIAD, University Bourgogne Franche-Comté, UTBM, 90010 Belfort, France; jean-charles.creput@utbm.fr

**Keywords:** normalized cross-correlation, Marr wavelets, entropy and response, graph matching, RANSAC

## Abstract

Automatically finding correspondences between object features in images is of main interest for several applications, as object detection and tracking, identification, registration, and many derived tasks. In this paper, we address feature correspondence within the general framework of graph matching optimization and with the principal aim to contribute. We proposed two optimized algorithms: first-order and second-order for graph matching. On the one hand, a first-order normalized cross-correlation (NCC) based graph matching algorithm using entropy and response through Marr wavelets within the scale-interaction method is proposed. First, we proposed a new automatic feature detection processing by using Marr wavelets within the scale-interaction method. Second, feature extraction is executed under the mesh division strategy and entropy algorithm, accompanied by the assessment of the distribution criterion. Image matching is achieved by the nearest neighbor search with normalized cross-correlation similarity measurement to perform coarse matching on feature points set. As to the matching points filtering part, the Random Sample Consensus Algorithm (RANSAC) removes outliers correspondences. One the other hand, a second-order NCC based graph matching algorithm is presented. This algorithm is an integer quadratic programming (IQP) graph matching problem, which is implemented in Matlab. It allows developing and comparing many algorithms based on a common evaluation platform, sharing input data, and a customizable affinity matrix and matching list of candidate solution pairs as input data. Experimental results demonstrate the improvements of these algorithms concerning matching recall and accuracy compared with other algorithms.

## 1. Introduction

Computer vision is an important research direction in current computer science since it occupies a pivotal position in human perception simulation. Automatically finding correspondences between object features in images is of interest for several applications, as target tracking [1], 3D object retrieval [2], pattern recognition [3], image stitching [4] and in many other fields.

Image matching is used to determine the geometric alignment of two or more images of the same scene taken by the same or different sensors from different viewpoints at the same or different times. We can distinguish dense correspondence, that determines correspondences at the pixel level, and sparse correspondence, that determines correspondences between a sparse set of higher lever features being first extracting from images. Most of the time such features represent invariant information at some location in the image, like corners, edges, gradients. Since we are interested in sparse correspondence, we study the standard methods for automatic extraction of the feature point sets from images. This becomes a critical step since it should avoid the presence of outliers and should allow discriminating objects easily. We propose a method for feature extraction step suitable to first-order matching.

Local feature descriptors, that is, providing detail feature detection and feature description information, play a fundamental and vital role in the process of feature correspondence, directly affecting the accuracy and objective score of graph matching (GM). High-quality local feature descriptors describe key points with uniqueness, repeatability, accuracy, compactness, and effective representation. These key points can keep robust and constant in terms of scaling, rotation, affine transformation, illumination, and occlusion [5]. Here we focus on the theoretical and mathematical descriptions of various local feature descriptors. In the feature points detection algorithm, local descriptors are typically used to describe image regions near feature points. Currently, methods for extracting feature points include Harris descriptor [6], Gilles descriptor [7], LoG descriptor [8], corner detector (CD) [9], Harrislaplace descriptor [10], SIFT descriptor [11], and so on. Among these feature extraction algorithms proposed in the literature, Marr wavelets which was originally used in [12] is favored for several properties: robustness (against distortion), rotationally invariant, noise insensitivity [13]. We choose to focus on the latter, whereas other methods will serve as a basis for comparative evaluation.

Given a pair of images, how to detect and extract feature points is the first step in image matching. Automatic feature extraction is the key point, and further related image matching can be performed based on the feature-to-feature correspondence. Given some standard nearest neighbor matching strategies, how to improve the reliability of the feature set is the problem to be solved here. To this end, we try to combine or enhance standard and easy-to-implement feature detection methods to make the final overall method (including feature detection and matching) competitive in terms of computation time and matching quality.

In order to obtain better matching results, the method proposed inserts a Laplace filter-based image preprocessing method before detecting feature points to increase the size of the candidate feature set. Since the Marr wavelet within scale-interaction method is more inclined to extract the edge information of the object, the Laplacian method can be used to enhance the edge details of the image. Then, a sparse feature point method based on entropy selection is proposed as a new filtering step. Filtering (also known as convolution) is also a very popular operation in the field of image processing, which can be applied to image encryption by changing pixel values [14]. This step combines the local entropy evaluation with the brightness deviation response as a new process for feature selection.

Entropy is a key concept in thermodynamics and statistical mechanics. It not only plays a special role in physical quantities, but also relates to the macro and micro aspects of nature, and determines the behavior of the macro system. Entropy is a well-defined quantity regardless of the type or size of the system under consideration. Entropy has many general properties, for instance, invariance, continuity, additivity, concavity, etc. [15].The probability distribution of entropy can be interpreted not only as a measure of uncertainty, but also as a measure of information [16]. Local entropy represents structured information, which is used to count the probability of occurrence of gray level in the sub-image, but independently around a single pixel. We claim that it is not sensitive to the influence of noise and can improve the accuracy of the image description. Based on the mesh division, the feature points in the sub-regions can be sorted according to their local entropy and selected trough deviation values. Then, entropy selection can not only effectively reduce the useless feature points for saving time, but also ensure the uniformity of feature point distribution. Mainly, the advantage of the proposed entropy and response algorithm is to realize a good compromise (trade-off) between accuracy and computation time together when compared to other standard approaches from literature. It is worth noting that both accuracy and computation time are essential criteria to compare and evaluate heuristic methods [17,18].

A necessary graph matching procedure based on the normalized cross-correlation similarity measure is applied to measure the effectiveness of the method in image matching. It entrusts quality assessment to the Random Sample Consensus Algorithm (RANSAC) program, which eliminates mismatched pairs and calculates true match recalls. The experiments were performed on standard image processing benchmarks. They showed how to increase the size of the feature set and matching accuracy with saving computation time.

The contributions of our paper are proposing two optimized algorithms: first-order and second-order for graph matching. Both of them are realized based on normalized cross-correlation (NCC) algorithm, which allows us to address graph matching or derived sub-problems with a closer relationship with experiments on integer quadratic programming (IQP) models in the Matlab platform. Especially for the first-order graph matching, we have proposed a new combination of feature points detection algorithms among Laplace filter, Marr wavelets, and the entropy-response based selection  method.

This paper is organized as follows. Section 2 introduces the motivations and taxonomy of graph matching. The formulation of standard graph matching is explained in Section 3. In Section 4, the different steps of the proposed feature extraction and first-order graph matching procedures are respectively presented. A preliminary version of this section forward appears as part of our previous conference paper [19]. We also extended the NCC based second-order graph matching in Section 5. The evaluation of the two proposed algorithms and their comparison with some algorithms are exposed in Section 6. Section 7 presents the conclusion.

## 2. Background of Graph Matching

### 2.1. Motivations of Graph Matching

Graph matching plays an important role in processing many practical applications in computer vision, such as feature correspondence [20], action recognition [21], image classification [22,23], shape matching [24], image retrieval [25], and pattern recognition [26,27]. The goal of graph matching is to find the optimal mapping constraint between two sets of nodes in two corresponding images, which will preserve the relationship between the graphs as much as possible so that when vertices are labeled based on the correspondence, they look ‘the most similar’.

In a more general case, this problem is expressed mathematically as a quadratic distribution problem, including finding a distribution that maximizes the objective function. Over the past decade, considerable effort has been invested in developing approximate methods to address more general QAP. Gold and Rangarajan [28] proposed a graduated assignment algorithm that combines graduated nonconvexity, two-way (assignment) constraints, and sparsity to solve a series of linear approximations to the cost function using Taylor expansion iterations. Leordeanu [29] presented an efficient approximation by using the spectral matching method (SM), which approximates the IQP problem to spectral relaxation. Cour et al. [17] introduced a new spectral matching with affine constraints (SMAC), which can not only provide a higher relaxation than SM but also keep the speed and scalability benefits of SM. Torresani et al. [20] designed a complex objective function which refers to dual decomposition (DD) that can be effectively optimized by double decomposition. Cho and Lee [18] presented reweighted random walk (RRWM) algorithms for graph matching. Later, Cho et al. [30] provided a max-pooling graph matching method (MPM), which not only resists deformation but also significantly tolerates outliers. This central idea of this algorithm is that the pairs with maximum scores are the correct matches.

Recently, some authors have proposed the use of high-dimensional relationships between super edges for high-order graph matching. The most commonly used is based on third-order research. The calculation of the high-order affinity matrix is generally based on the tuples of feature points, and it is achieved by comparing the corresponding edges and angle information of two sets of corresponding triangles. Another vital characteristic of high-order matching is that it is invariant to changes in scale and affine. Zass and Shashua [31] reformulated a high-order graph matching (HGM) in the view of probabilistic view of the probability setting of convex optimization representation. Chertok and Keller [32] proposed a general framework for solving higher-order assignment problems based on the core assumption that high order affinities are encoded in an affinity tensor. In this algorithm, they derive a marginalization scheme that can map triples to matrices or vectors. Olivier Duchenne et al. [33] derived a tensor-based high-order graph matching (TM) that invariant to affine, rigid, or transformations. This algorithm defined a tensor to represent the affinity assignment between the tuples of features. It is a multidimensional power iteration operation during which the solution will be projected onto the closest assignment matrix. Lee et al. [34] demonstrated a hypergraph matching via reweighted random walks (RRWHM) in a probabilistic manner. The algorithm uses a personalized jump and re-weighting scheme, which effectively reflects the one-to-one matching constraints in the random walk process. It can realize strong anti-deformation and anti-noise performance compared with other state-of-the-art methods. Ngoc [35] presented a general framework with a flexible tensor block coordinate ascent scheme for hypergraph matching. It is a crucial idea under a multilinear reconstruction using the original objective function, which can guarantee the third-order matching scores their algorithms increase monotonically. Another hypergraph matching algorithm based on tensor refining was proposed in [36], accompanied by an alternative adjustment approach to accelerate the convergence processing.

Despite decades of extensive research, graph matching is still a challenging problem for two main reasons: (1) generally, the objective function is non-convex and prone to local minima. (2) The constraints of space and time complexities. We set out to conduct research to solve these challenges.

### 2.2. Taxonomy of Graph Matching

The goal of the graph matching process is mainly focused on finding the correspondences between two characteristics, edges, and points, under certain constraints. A graph-based matching method treats a set of points as graphics. In the most common cases, the algorithm used to solve inexact matching problems can be classified into three categories: first-order, second-order, and high-order graph matching methods.

- First-order graph matching At the view of first-order graph matching, it mainly implicates vertex-to-vertex properties based on local feature descriptors, focuses primarily on unary information, regardless of edge-related associated information. This matching method was proposed initially to find the correspondence between two sets of points and transformation parameters at the same time. It uses a coordinate positioning and grayscale information to calculate the transformation parameters and uses a soft assignment algorithm to estimate the correspondence between two sets of points. Finally, it converts the alignment of the two-point sets into the optimal match between the two graphs. Although the result of the first-order matching is stable, it fails when there is ambiguity, such as local appearance and repeating texture. In the case of high noise and outlier registration, it performs poorly, which dramatically limits its range of applications. Therefore, first-order matching is more suitable for focusing on non-rigid motion types with a small local affine transformation.
- Second-order graph matching Second-order graph matching mainly enriches the vertex, and edge approaches; it is objective function established by a matrix representing the affinity properties between candidate pairs, that is to say, each node represents the correspondence between points, and the weights represent pairwise protocols between the corresponding potential pairs. A secure connection of the adjacency matrix can judge the correct assignment or not. Therefore, it better stands for some point of view between candidate pairs and overcomes the disadvantages of the first-order algorithm. Since the graph matching algorithm is usually based on the integer quadratic programming (IQP) formula, with an approximate solution, so the second-order graph matching problem is also the NP-hard problem. That means it can be formulated as an optimization problem; the purpose is to get the best match and receive a higher score based on the objective scoring function.
- High-order graph matching Hypergraph is a natural generalization of traditional graphs. Since pairwise assignments are sensitive to scale-invariant between two corresponding graphs, pairwise relationships are not enough to capture the entire geometrical structure. Unlike pairwise matching in which each link can have two vertices at its ends, each link in the hypergraph can have three or more vertices, which can have a more powerful tool to model complex structures for more high-level information. Therefore, the most crucial idea to solve the hypergraph problem is to search for higher-order constraints, rather than unary or pairwise constraints. Essentially, hypergraph matching is a combinatorial optimization problem. In the process of obtaining the final solution, it is not straightforward for us to find its optimal global solution based on a reasonable time. In recent years, more accessible methods use probability frames to explain hypergraph matching, of which tensor-based models are often used. Considering the complex structure of the data, we believe that the learning and construction of hypergraphs will hopefully become an increasingly promising research direction in the future.

## 3. General Formulation of Graph Matching Problem

This section introduces the general representation of traditional graph matching and the definition of the high-order graph matching problem. The main purpose is on finding the correspondence one-to-one mapping between two feature sets from two image sources. The goal is to maximize a function score among the set of correspondence pairs. In first-order matching, only local attribute descriptors are considered and evaluated, whereas, in the general case of graph matching, two order potentials between pairs (edges) of features must also be maximized to established the similarity between edges of features. On the other hand, the high-order GM method considers the invariant geometric information by considering the relationship between tuples of feature points. The input feature graph becomes a hyper-graph, where hyper-edges replace edges, that is subsets of *k* points, with the order k≥2, rather than only considering couple of points.

**Figure 1:**
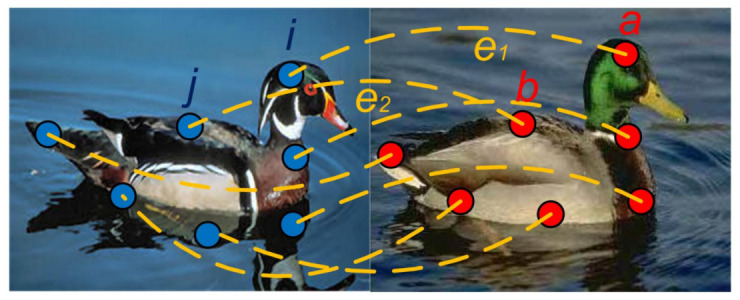
Feature point correspondence mapping.

Suppose we are given a pair of graphs $G_{P}=\left(P,E^{P}\right)$ with $N_{P}$ feature points for the reference graph $G_{P}$, and $G_{Q}=\left(Q,E^{Q}\right)$ with $N_{Q}$ feature points for the query graph $G_{Q}$. *P* and *Q* are the two sets of feature points, and $E^{P}$ and $E^{Q}$ denote edge sets. We note i,j∈P and a,b∈Q as representing feature points. Therefore, the main problem is to find a suitable one-to-one mapping from one feature set to the other feature set as illustrated in the Figure 1. The pictures in Figure 1 are from PF-WILLOW dataset (https://www.di.ens.fr/willow/research/proposalflow/).

Finding a mapping form *P* to *Q* can be equivalent to find an $N_{P}\times N_{Q}$ assignment matrix *X*, such that $X_{ia}=1$ when point *i* is assigned to point *a*, and $X_{ia}=0$ otherwise. Therefore, a one-to-one admissible solution must verify the following constraints in (1), that requires a binary solution, and (2) and (3), that express the two-ways constraints of a one-to-one mapping.

(1) $$X\in {\left\{0,1\right\}}^{N_{P}\times N_{Q}}$$

(2) $$\forall i\sum_{a=1}^{N_{P}}X_{ia}\leq 1$$

(3) $$\forall a\sum_{i=1}^{N_{Q}}X_{ia}\leq 1$$

Then, the problem of graph matching can be formulated as the maximization of the following general objective score function (4):

(4) $$score\left(X\right)=\sum_{ia,jb}H_{ia,jb}X_{ia}X_{jb},$$

where $H_{ia,jb}$ means the similarity or affinity measurement corresponding to the tuple of feature points i,j and a,b. The higher is the score $H_{ia,jb}$, the higher are the similarities between the two corresponding edges $\left(i,j\right)$ and $\left(a,b\right)$. The product $X_{ia}X_{jb}$ is equal to 1 if and only if points i,j are respectively mapped to points a,b.

Then, we need to know how to compute such a positive and symmetric similarity matrix *H*. Many cost functions may be used to compute affinity matrices for first-order and second-order GM. Note that $H_{ia,ia}$ represents first-order similarity term, between local attributes of points i∈P and a∈Q. For example, the authors in [37,38] use the normalized cross-correlation (NCC) cost function, as we have used to validate our feature point extraction method in this thesis. Nevertheless, any point-to-point distance function can be used, as the Euclidean distance between SIFT descriptors, or sum of squared error data terms.

Duchenne et al. in [33] propose a general formula to compute the second-order affinity term $H_{ia,jb}$ as shown in (5), where *f* is a feature vector associated to each edge.

(5) $$\forall ia,jb\;H_{ia,jb}=exp\left(-\gamma {∥f_{i,j}-f_{a,b}∥}^{2}\right)$$

Leordeanu et al. in [29], and as most often encountered, computes the Euclidean distance between the corresponding candidate point pairs i,j and a,b, to build the affinity term $H_{ia,jb}$, as shown in Equation (6). Here, $\sigma_{d}$ is the sensitive controller of the deformation.

(6) $$H\left(ia,jb\right)=\left\{\begin{array}{ccc} 4.5-\frac{{\left(d_{ij}-d_{ab}\right)}^{2}}{2\sigma_{d}^{2}} & \; & if|d_{ij}-d_{ab}|<3\sigma_{d}\\ 0 & \; & otherwise \end{array}\right.$$

## 4. First-Order NCC Based GM

**Figure 2:**
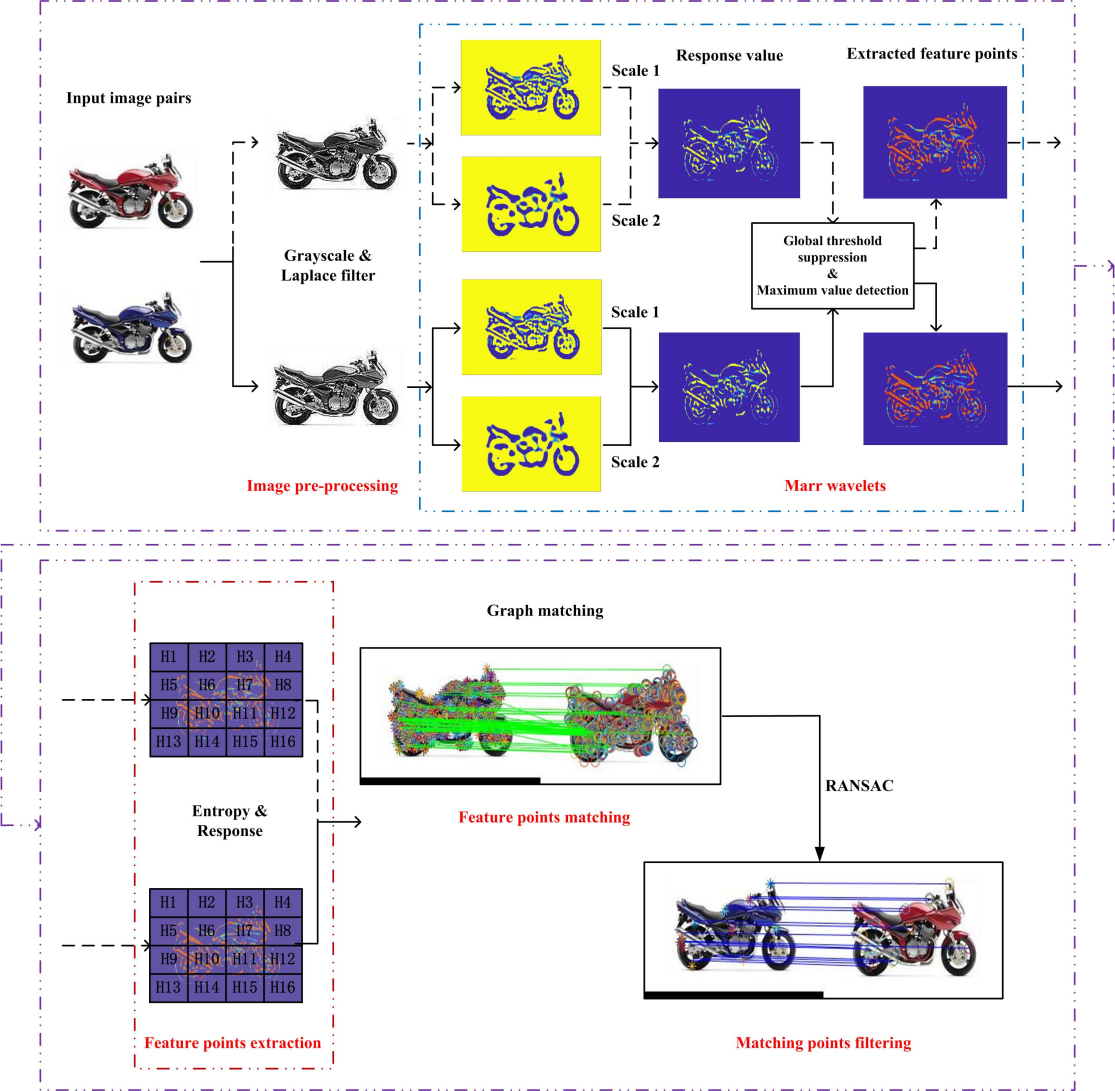
The basic flowchart of graph matching.

In this section, we introduce the implementation of the system image matching process, including feature point detection and extraction, feature point matching, and removing mismatching pairs. The schematic diagram of the whole process is shown in Figure 2. Regarding the interest point matching strategy, the proposed pipeline illustrated in Figure 2 can be summarized as (1) Laplacian filter is used for edge detection; (2) Marr wavelets are used to identify salient points in the image; (3) entropy based metric for selecting the most distinctive points of the image denoted as feature points; (4) feature points matching; (5) outlier removal through RANSAC.

### 4.1. Image Pre-Processing

Laplacian is a second-order derivative operator that detects the zero-crossing in image intensity and usually produces more accurate edge detection results [39]. Laplace filter represents a discrete approximation to the mathematical Laplace operator. Its second-order partial derivative in the orthogonal direction of continuous space and the approximation of its mathematical equivalent are defined below [40]:

(7) $$\nabla^{2}f\left(x,y\right)=\frac{\partial^{2}f\left(x,y\right)}{\partial x^{2}}+\frac{\partial^{2}f\left(x,y\right)}{\partial y^{2}}$$

(8) $$\nabla^{2}f\left(x,y\right)≅\left\{f\left(x+1,y\right)+f\left(x-1,y\right)+f\left(x,y+1\right)+f\left(x,y-1\right)\right\}-4f\left(x,y\right)$$

(9) $$I\left(X\right)=I\left(x,y\right)=f\left(x,y\right)-c\cdot \nabla^{2}f\left(x,y\right)$$

where f(x,y) is the original image, I(x,y) is the processed image, *c* is a constant.

**Figure 3:**
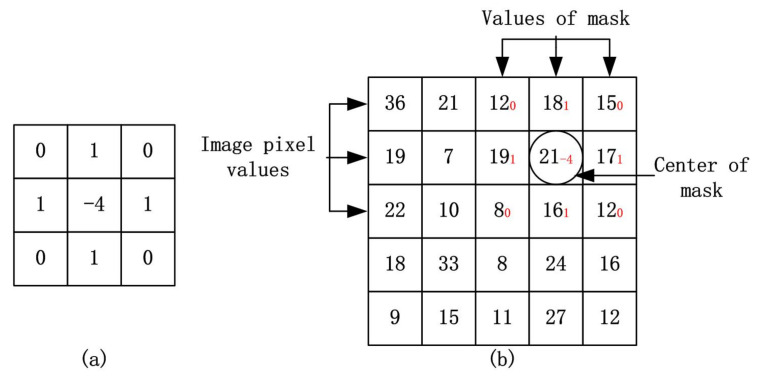
The process of the Laplacian filter sharpening: (**a**) the digital mask filter *w*, (**b**) the convolution operation.

From formula (8), the digital mask filter *w* can be viewed as the following 3×3 set of filter coefficients as shown in Figure 3a. The process of the Laplacian filter sharpening is essentially a convolution process. Suppose the origin pixel of *f* is located in the upper left corner of the image *f*, and set the middle value of mask *w* as the center of kernel. Let *w* move at all possible positions so that the center kernel of *w* can coincide with each of pixels of *f*. The convolution operation is essentially the sum of the products of the corresponding positions of the two functions. The convolution between *f* and its corresponding mask filter *w* is shown in Figure 3b.

The definition of two-dimensional convolution is as:

(10) $$I\left(x,y\right)=f*w=\sum_{k,l}f\left(x+k,y+l\right)w\left(k,l\right)$$

### 4.2. Marr Wavelets within Scale-Interaction

Receptive field [41] is used to describe the stimulation pattern of the retina. The receiving field of high-level neuron cells in the visual pathway is synthesized from the receiving field of low-level neuron cells. Therefore, as the level increases, the range of the receptive field becomes more impressive. Ultra-complex neuron cell models [42] can respond to complex object features through powerful non-linear processing functions, and most ultra-complex neuron cells have sensitive termination characteristics at the ends of line segments, corner points, and line segments with high curvature. In other words, the response of ultra-complex neuron cells to light can be simulated by the difference in response of spatial filters with different bandwidths to light. The response of the received field to light can be represented by a spatial filter function, such as a Gaussian difference function or a Gabor wavelet function.

The scale-interaction model for feature detection is based on filtering using a class of self-similar Gabor functions or Gabor wavelets [43,44], which can achieve the minimum joint resolution in the spatial and frequency domains. This recommendation is made because it is unique in reaching the smallest possible value of the joint uncertainty [45]. The function of feature detection is defined as the following formula:

(11) $$Q_{ij}\left(x,y,\theta \right)=f\left(W_{i}\left(x,y,\theta \right)-\gamma W_{j}\left(x,y,\theta \right)\right)$$

where γ is a normalizing factor, $W_{i}\left(x,y,\theta \right)$ and $W_{j}\left(x,y,\theta \right)$ are spatial filters. They go through the transformation of a nonlinear function *f* at location (x,y) with preferred orientation θ in two scales *i* and *j* respectively. If feature detection function $Q_{ij}$ obtains a local maximum at the location (x,y), this location is considered to be a potential feature point position.

**Figure 4:**
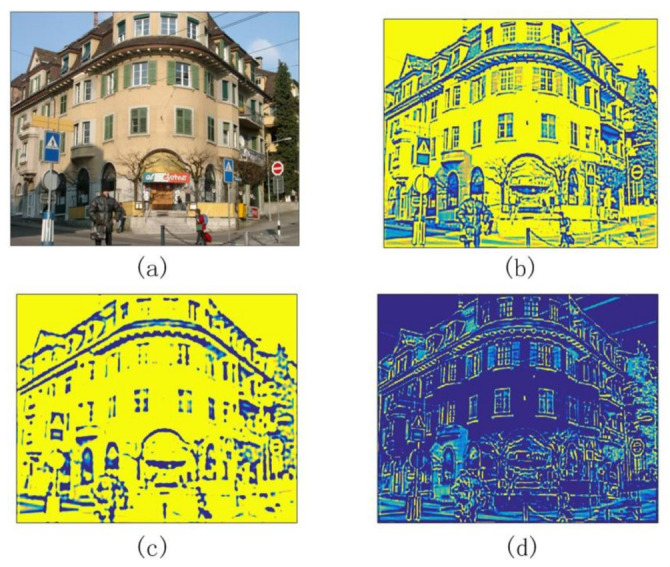
From the left to the right: (**a**) original image, (**b**) filtered result when i=1, (**c**) filtered result when i=2 and (**d**) response image.

For further optimization, the Marr wavelets [46] were used instead of Gabor wavelets within the scale-interaction model, because of its isotropic [47,48]. Two-dimensional Marr wavelets and their corresponding feature detection function are defined as:

(12) $$M_{i}\left(X\right)=\lambda_{i}\left(2-\lambda_{i}^{2}X^{2}\right)exp\left(-\frac{\lambda_{i}^{2}X^{2}}{2}\right)$$

(13) $$Q_{ij}\left(X\right)=\left|M_{i}\left(X\right)-\gamma M_{j}\left(X\right)\right|$$

(14) $$R_{ij}\left(X\right)=I\left(X\right)*Q_{ij}\left(X\right)$$

where X=(x,y), and $X^{2}=\left(x^{2}+y^{2}\right)$. $\lambda_{i}=2^{-i}$ and *i* represents the scaling value of Marr wavelets. Convolve $Q_{ij}\left(X\right)$ with an grayscale image I(X). If its response value $R_{ij}\left(X\right)$ obtains a maximum local value, then *X* is considered to be a potential feature point. Algorithm 1 is pseudo-code of Marr wavelets within scale-interaction. Figure 4 illustrates the process of extracting response value using Marr wavelets within scale-interaction. (b) and (c) are convolution results between the original picture and the mask filter. Then local maximum points are extracted on this basis.
**Algorithm 1** Marr wavelets within scale-interaction algorithm**Input:**
 I(X),i,j**Output:** points  1:
$I_{i}=MarrFilter\left(I\left(X\right),i\right)$
  2:
$I_{j}=MarrFilter\left(I\left(X\right),j\right)$
  3:
$I_{sub}\leftarrow \left|I_{i}-I_{j}\right|$
  4:
$local_{thr}\leftarrow max\left(max\left(I_{sub}\right)\right)*r$
  5:
 **if**
$I_{sub}\left(i,j\right)<local_{thr}$
 **then**
  6:    $I_{localthr}\left(i,j\right)\leftarrow I_{sub}\left(i,j\right)\leftarrow 0$
  7:
**end if**
  8:
$points\leftarrow corner_{peaks}\leftarrow I_{localthr}$
  9: **function** MarrFilter(I(X),scale) 10:    $\delta =2^{scale}$ 11:    x=−(2*fix(δ)):1:(2*fix(δ)) 12:    y=−(2*fix(2*δ)):1:(2*fix(2*δ)) 13:    $M_{i}\left(X\right)\leftarrow X\leftarrow meshgrid\left(x,y\right)$ 14:    $I_{fil}\leftarrow I\left(X\right)*M_{i}\left(X\right)$ 15:
**end function**


### 4.3. Entropy and Response

Aiming at the problems of uneven feature distribution, too many feature points, and long matching time, a feature point extraction method based on local entropy and feature point response was proposed, called the entropy and response algorithm (ER). In this section, three main parts are explained.

#### 4.3.1. Entropy Algorithm

In actual images, feature points often appear as sharp changes of gray values or inhomogeneity in grayscale distribution; that is, the local region of a feature point has a large amount of information. Entropy is a measure of information in an image, and local entropy is a measure of local area information of an image. Local entropy value under feature-rich region is much higher than the local entropy value under feature-poor region. Therefore, it is possible to determine which regions have more features by calculating the local information entropy of image, and then extract the feature points in these regions.

**Figure 5:**
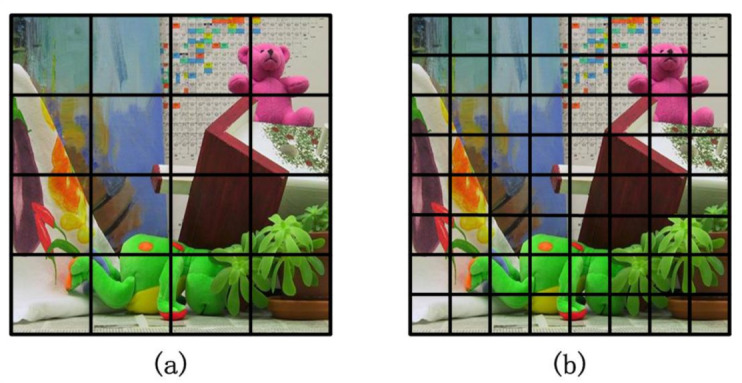
Schematic diagram of meshing: (**a**) image divided by 4×4 sub-regions, (**b**) image divided by 8×8 sub-regions.

Information entropy [49,50] is the amount that represents the overall characteristics of the source in a common sense. It is considered from the statistical properties of the entire source to measure the expected value of a random variable. An image is essentially a source of information that can be described by information entropy. Let the gray image *G* have *m* gray levels, mesh division is performed to obtain n×n sub-regions. The whole information entropy $H_{i}$ and the average entropy $\bar{H}$ of the image are calculated as follows:

(15) $$H_{i}=\sum_{i=1}^{m}p_{i}log_{2}p_{i}$$

(16) $$\bar{H}=\frac{1}{n^{2}}\sum_{j=1}^{n^{2}}H_{j}$$

where $p_{i}$ is the probability that the $i_{th}$ gray level appears, that is, the ratio of the number of pixels whose gray value is *i* to the total number of pixels of the image. So the local entropy is counted for the probability of occurrence of gray level in the sub-image. Since the value of the information entropy is only related to the distribution of the local gray-scale pixels, but independent with a single pixel, so it is not sensitive to the influence of noise and can improve the accuracy of the image authenticity description. Here, we use the local information entropy of the image to extract feature points. Under the meshing strategy, the image is divided into n×n sub-regions. Therefore, the sub-average entropy value of each sub-grid can be calculated. Figure 5 is a schematic diagram of mesh division, *n* is set to 40.

#### 4.3.2. Response Algorithm

After mesh division and the computation of each local entropy, we will get n×n sub-regions for the whole image, then detected feature points are mapped into the respective sub-areas. In this case, if we compute and sort the entropy values of all of these feature points extracted, then the first *N* feature points with larger entropy values can be selected to describe the whole image. However, this method only utilizes the entropy values of feature points, without considering its distribution in the image. Finally, feature points with high entropy values may mostly appear in the same local area, which will cause aggregation. So a block division and response algorithm are proposed to deal with this problem.

**Figure 6:**
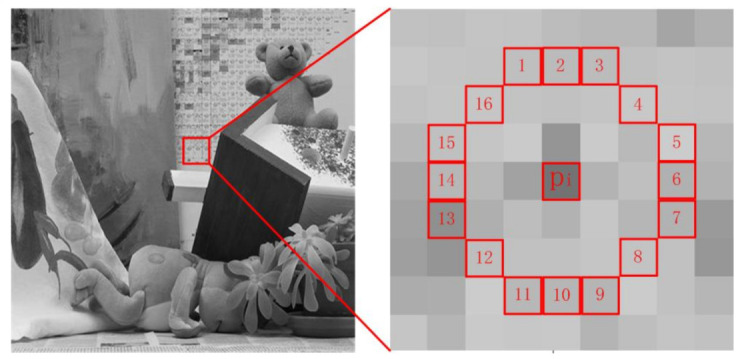
Bresenham discrete circle centered on pixel $p_{i}$.

As mentioned before, if a pixel presents a sharp change in its neighborhood, this pixel will have a stronger deviation value from the mean value. Based on the Bresenham discrete circle [51] with the pixel point $p_{i}$ as the center and three pixels as the radius, 16-pixel points on the discrete circumference are considered in correspondence with the central pixel point $p_{i}$. This is shown in Figure 6. These 16 pixels are assigned to dark and bright areas. The dividing criteria and deviation [52] are respectively defined as the following:

(17) $$S_{bright}=\left\{x|I_{p_{i},x}>I_{p_{i}}+t\right\}$$

(18) $$S_{dark}=\left\{x|I_{p_{i},x}\leq I_{p_{i}}-t\right\}$$

(19) $$Dev=max(\sum_{x\in S_{bright}}|I_{p_{i},x}-I_{p_{i}}|-t,\sum_{x\in S_{dark}}|I_{p_{i}}-I_{p_{i},x}|-t)$$

where $S_{bright}$ indicates bright area, $S_{dark}$ indicates dark area. $I_{p_{i}}$ is the gray value of center point $p_{i}$, $I_{p_{i},x}$ represents the gray value of a pixel labeled *x* on a discrete circumference centered at pixel $p_{i}$, *t* is the set threshold. Dev is the sum of the deviation value among the gray values of the pixel $p_{i}$ and its corresponding neighboring pixels located in the bright or dark area.

#### 4.3.3. Distribution Criterion

After the calculation of the entropy and response, a distribution criterion is proposed to extract the corresponding points that meet the requirements. In the region where the local entropy is bigger than the average of entropy $\bar{H}$, the feature points are extracted with the ratio *r*. The only one strongest response point is extracted in each remaining region where the local entropy is smaller than $\bar{H}$. Here, we choose the unified ratio method for the selection of *r*. Assuming that there are *m* regions whose local entropy is greater than $\bar{H}$, then $r_{i}\left(1,2,\ldots ,m\right)$ is the ratio of extracting feature points in the $i_{th}$ region. For example, when r=10%, that is, in the $m_{th}$ region with large local entropy, the feature points with the top 10% responses given by Dev are extracted. The appropriate value of *r* is set empirically.

The detail of the entire ER method is outlined in Algorithm 2. Based on the mesh division strategy, we first compute each of the local average entropy value of all these sub-regions. Then the feature points are sorted according to the computation of their deviation values in each of their sub-region. Finally, the distribution criterion can not only effectively reduce some of the useless feature points, but also ensure the uniformity of feature point distribution. As we can see, the step entropy and response can both develop a custom algorithm to identify feature points. Entropy strategy is only to calculate the reflection degree of an individual pixel, but the response is based on the deviation value of a set pixels between the center point, and it is adjacent points based on the Bresenham discrete circle principle, so that the mutation of response is more reflected for finding points with reliable contrast. Therefore, deviation is more capable of extracting more qualified feature points than entropy.
**Algorithm 2** Entropy and response algorithms**Input:** 
I(X),points,m,n,t,r**Output:** 
$points_{select}$  1:  **for** 
$j=1\to n^{2}$ 
**do**
  2:    $points_{j}\leftarrow points$
  3:    $H_{j},\bar{H}\leftarrow Entropy\left(I\left(X\right),m,n\right)$
  4:    $p_{sub(j)}\leftarrow Response\left(points_{j},t\right)$
  5:    **if**
$H_{j}⩾\bar{H}$
**then**
  6:        $points_{select}\leftarrow p_{(p_{sub(j)}*r})$
  7:
    **else**
  8:        $points_{select}\leftarrow p_{max}$
  9:    **end if** 10:    j=j+1 11: **end for** 12:
**return** 
$points_{select}$ 13: **function** Entropy(I(X),m,n) 14:    $p_{i(m*1)}\leftarrow Isub{\left(i\right)}_{\left(n*n\right)}\leftarrow I\left(X\right)$ 15:    $H=\sum_{i=1}^{m}p_{i}log_{2}p_{i}$
 16:    $\bar{H}=\frac{1}{n^{2}}\sum_{j=1}^{n^{2}}H_{j}$
 17:
**end function**
 18: **function** Response(points,t)
 19:    $I_{p,x}\leftarrow circle_{p}\leftarrow points$
 20:    $Dev=max(\sum_{x\in S_{bright}}|I_{p,x}-I_{p}|-t,\sum_{x\in S_{dark}}|I_{p}-I_{p,x}|-t)$
 21:    $p_{sub}\leftarrow p_{\left(Dev_{p}\right)}$
 22:
**end function**


### 4.4. Definition of First-Order GM Problem Based on NCC

The purpose of graph matching [53] is to determine the correct attribute correspondences $P=(V^{P},E^{P})$ and $Q=(V^{Q},E^{Q})$ between two graphs *P* and *Q*, where *V* means vertex, and *E* represents edge. We customize corresponding mapping edges $e_{1}=ij\in E^{P}$, $e_{2}=ab\in E^{Q}$.

The objective of graph matching is to find the correct corresponding point pairs between two graphs *P* and *Q* among the feature points extracted. A unidirectional ’one-to-one’ constraint is assumed, which requires one node in *P* to match at most one node in *Q*.

Cross-correlation is a standard method for estimating the similarity between two sets of data [54]. Normalized cross-correlation (NCC) is an essential application, which has been used widely for many signal processing applications due to its effective and direct representation in the frequency domain, and it is less sensitive to linear variations in the amplitude of two comparison signals [55]. We use the NCC algorithm to measure the similarity between two feature point *p* in graph *P* and *q* in graph *Q*. These ratios Ra(p,q) of the calculated correlation values represent the degree of matching between the two sets of corresponding images. The NCC algorithm used to find similarity match between a window near feature point *p* and a window around feature point *q* is defined as:

(20) $$Ra\left(p,q\right)=\frac{\sum_{i}\left[\left(Wp_{i}-\bar{Wp}\right)\left(Wq_{i}-\bar{Wq}\right)\right]}{\sqrt{\sum_{i}{\left(Wp_{i}-\bar{Wp}\right)}^{2}}\sqrt{\sum_{i}{\left(Wq_{i}-\bar{Wq}\right)}^{2}}}$$

where the summations are over all window coordinates, $Wp_{i}$ and $Wq_{i}$ are pixel intensity in windows for *p* and *q* respectively, each of the windows is sized as 5×5. Also, $\bar{Wp}$ and $\bar{Wq}$ are the corresponding mean of the window pixels. The coordinate of maximum values in this normalization cross-correlation is the position of the best matches for reference images.

**Figure 7:**
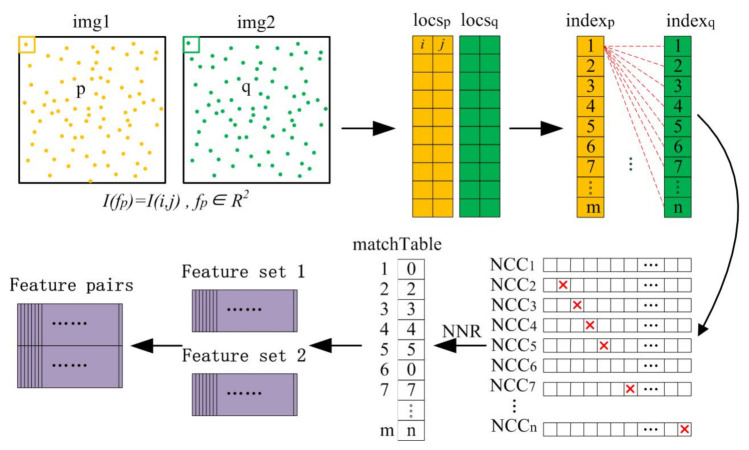
The basic flowchart of data processing.

Based on the NCC similarity measure, we use the nearest neighbor ratio (NNR) method to perform a rough match on the feature point set. The selection and matching process of the NNR algorithm with one-way ‘one-to-one’ constraint is described as follows: Based on the sample feature point pairs in the two images, first use the NCC algorithm to extract all the most significant corresponding pairs. These ratios are then compared with a fixed threshold. If the NCC ratio is higher than this fixed threshold, the corresponding point pair is considered a match. Otherwise, the pair of points are discarded. The fixed threshold is usually a constant not greater than 0.9. Since correct matching has stronger similarity than incorrect matching, this is a functional judging characterization for graph matching according to the NNR concept. Figure 7 shows a flowchart of data processing. The detected feature points of the two images are respectively put into two corresponding buffers. Each point *p* in graph *P* is used to calculate the ratio of NCC to all points *q* in graph *Q*, and then all ratios are sorted in descending order. Under the principle of NNR, some will be extracted as the best matching points, otherwise, they will be discarded.

### 4.5. Outlier Elimination

In this application, before using the Marr filter for convolution, we will use Laplacian for convolution as a preprocessing step. Due to the linear relationship of these two operations, this corresponds to a single convolution, which is a smoothed fourth-order derivative filter. Therefore, as each derivative amplifies the noise, the number of feature points increases. On the other hand, although the NNR method is easy to implement and sometimes well-matched, some points in these extracted feature point sets do not match, so a mismatched cleanup operation is required. Therefore, it is particularly important to find ways to reduce the mismatch caused by interference.

The RANSAC algorithm [56,57] is called the Random Sample Consensus Algorithm, which can well eliminate the existence of mismatches. The algorithm has strong robustness and the ability to correct data sets. The basic idea of the RANSAC algorithm is to use an iterative method to extract the sample set from the model. Find an optimized parametric model that can include more internal points in the data set and then test the extracted samples using the residual set. Points in the algorithm that fit the data set model are called interior points. Otherwise, they are called outliers. Therefore, the RANSAC algorithm can be used to find the best parameter model in the data set containing outliers through an iterative algorithm. The detailed implementation process of RANSAC is as follows:

(a) Randomly extracts non-collinear *a* pairs of feature points from the data set (a=4 in experiment), then calculate their transformation homography matrix *H*, and record it as model *M*.
(b) Calculate projection error of each point in the dataset with model $M_{k}$. If the error is less than a predefined threshold τ, add it into the inner point set $I_{k}$.
(c) If the current number of elements in inner point set $I_{k}$ is greater than the number in optimal inner point set $I_{best}$, then update $I_{best}$ and re-estimate the model $M_{best}$.
(d) If the number of iteration is more than *k*, the operation will be exited; otherwise, the number of iterations is increased by 1, and the above steps are repeated.

The threshold τ is selected in accordance with *n*-dimensional chi-square distribution. χ is the cumulative chi-square distribution. Assume that the out-of-class points are white Gaussian noise with a mean of 0 and a variance of η. The number of iterations *k* will be updated instead of fixed until it is greater than the maximum number of iterations.

(21) $$\tau^{2}=\chi_{n}^{-1}\left(\mu \right)\eta^{2}$$

(22) $$k=\frac{log(1-p_{c})}{log(1-\mu^{a})}$$

where $p_{c}$ is the confidence level, generally taking 0.95 to 0.99; μ is the ratio of inlier point; *a* is the minimum number of samples required to calculate for model. The pseudo-code of RANSAC is outlined in Algorithm 3.
**Algorithm 3 **Random Sample Consensus (RANSAC) algorithm**Input:** 
$p_{c},k_{max},\tau ,a,m$**Output:** 
$M_{best},I_{best}$  1:
$k=0,I_{max}=0$
  2:
**for** 
$k<k_{max}$
**do**
  3:    $\tau^{2}=X_{n}^{-1}\left(\mu \right)\eta^{2}$
  4:    Use randomly sampled subset *a* to estimate $M_{k}$ and $I_{k}$
  5:    **if**
$|I_{k}|>I_{max}$
**then**
  6:        $M_{best}=M_{k},I_{best}=I_{k}$
  7:        $\mu =|I_{best}|/m,k_{max}=log\left(1-p_{c}\right)/log\left(1-\mu^{a}\right)$
  8:    **end if**
  9:    k=k+1 10:
**end for**

### 4.6. Parameters of the Proposed Algorithm

This section details the specific parameters used in the experiments. In the feature point detection part, the Marr wavelet algorithm is used to define the feature points as the maximum local value inside the scale-interaction image (with γ=1). The two scales we chose are i=1 and j=2. Then the mesh division and feature points extraction are processed, the image is meshed by n×n to obtain $n^{2}$ sub-regions. Here, n=40 is selected. The detected feature points are mapped into various sub-regions and sorted based on the deviation value Dev in each sub-region to which they belong. At the same time, each local information entropy $H_{i}$ and average information entropy $\bar{H}$ are calculated. Assume that there are *k* sub-regions with local information entropy greater than the average information entropy, then feature points with maximum responsiveness of 30% are extracted from these *k* regions. In the feature point matching part, the NCC similarity measurement algorithm and NNR method are used to match the feature point set roughly. For the matching point filtering part, RANSAC can better remove the unmatched points, and finally get more accurate matching results. Its computational complexity is $O\left(max(N_{P},N_{Q})\right)$, where $N_{P}$ is the number of features in the reference image, and $N_{Q}$ is number of features in query image. The algorithm is suitable for performing real-time global methods. Besides, it can also achieve efficient parallel implementation in GPU systems. Algorithm 4 outlines the details of the entire process of the proposed algorithm.
**Algorithm 4** The proposed algorithm**Input:** Input images $I_{1}$ and $I_{2}$
1:$I_{1}$ and $I_{2}$ processed under Laplace filter2:$\left(I_{1^{\prime }},I_{2^{\prime }}\right)=MarrWaveletsFunction\left(I_{1},I_{2}\right)$3:$\left(points1,points2\right)\leftarrow \left(corner_{peaks1},corner_{peaks2}\right)\leftarrow \left(I_{1^{\prime }},I_{2^{\prime }}\right)$4:$\left(points_{select1},points_{select2}\right)=EntropyResponseFunction\left(points1,points2\right)$5:$\left(points_{select1^{\prime }},points_{select2^{\prime }}\right)=RANSACFunction\left(points_{select1},points_{select2}\right)$6:$MatchingPairs=NCCandNNR(points_{select1^{\prime }},points_{select2^{\prime }})$


## 5. Second-Order NCC Based GM

The first-order GM provides convolution-based algorithms, whereas the second-order GM emphasizes geometric inter-feature relationships, transforming the correspondence problem to a purely geometric problem stated in a high dimensional space, generally modeled as an integer quadratic programming. This section presents our second application. We introduce in this section a new contribution with an application to second-order graph matching in the Matlab framework. The framework is based on the original Matlab application provided by Cho et al. [18]. This application constitutes a useful framework for graph matching as an IQP problem. It offers useful mathematical abstractions, and it allows us to develop and compare many algorithms based on a common evaluation platform, sharing input data, but also customizing affinity matrices and a matching list of candidate solution pairs as input data. This allows us to reuse these common data and context to start elaborate NCC algorithms for second-order graph matching application (As we discussed in detail in Section 4.4). This approach uses NCC algorithm to search for the indicator vector, then the matching score will be computed under IQP based formulation. By considering the second-order term, the algorithm determines the mapping between two graphs that should reflect the geometric similarity relationship between the pairwise matching features. All the algorithms are executed and compared based on the same experimental framework with common data from standard benchmarks in the domain.

We set *P* and *Q* the two sets of features of query graph $G_{P}=\left(P,E^{P}\right)$ and reference graph $G_{Q}=\left(Q,E^{Q}\right)$ respectively. We note i,j∈P and a,b∈Q as feature points, $ij\in E^{P}$ and $ab\in E^{Q}$ as edges. Also, $e_{1}=\left(i,a\right)$ and $e_{2}=\left(j,b\right)$ represent, when needed, candidate assignments. The main task it to find a suitable one-to-one mapping between *P* and *Q*. The feature points correspondence mapping is shown in the Figure 1. The yellow lines are correct matches.

Affinity matrix *M*, also known as affinity tensor, is used to organize the mutual similarities between sets of feature points. The measurement of affinity can be interpreted as a product of a solution vector *x*, that represents the set of candidate correspondences, by the matrix. The solution variable $x\in {\left\{0,1\right\}}^{N_{P}N_{Q}}$ is an indicator vector such that $x_{ia}=1$ means feature i∈P matches with feature a∈Q, $x_{ia}=0$ means no correspondence, and where $N_{P}$ and $N_{Q}$ are the respective set sizes of *P* and *Q*.

A graph matching score *S* between edges can be defined by the following equation:

(23) $$S=\sum_{ij∼ab}f\left(ij,ab\right)=\sum_{ia,jb}M\left(ia,jb\right)x_{ia}x_{jb}=x^{T}Mx,$$

where ij∼ab means (i,a) and (j,b) are correspondence pairs, and *x* is the indicator vector. Then, the purpose of the graph matching IQP problem is computing solution $x^{*}$ that maximizes the matching score as follows:

(24) $$\begin{array}{c} x^{*}=argmax\left(x^{T}Mx\right), \end{array}$$

(25) $$s.t.\;x\in {\left\{0,1\right\}}^{N_{P}N_{Q}},$$

(26) $$\forall i\sum_{a=1}^{N_{Q}}x_{ia}\leq 1,$$

(27) $$\forall a\sum_{i=1}^{N_{P}}x_{ia}\leq 1.$$

The binary constraint is expressed by Equation (25), while (26) and (27) express the two-way constraints, that specify the solution to be a one-to-one mapping from *P* to *Q*. Note that by removing constraint (27), we obtain a many-to-one mapping, that is, a (partial) function from *P* to *Q*. In this chapter both constraints must be verified.

Affinity matrix *M* which consists of the relational similarity values between edges and nodes must is considered as an input of the problem. It can be noted that its size is defined by the total number of candidate assignment pairs considered. Then, the affinity matrix size may vary from $O\left({\left(N_{P}N_{Q}\right)}^{2}\right)$, in the case of full possible pairs, to $O\left({\left(K\times N_{P}\right)}^{2}\right)$ where *K* is some constant, in case of a restricted list of candidate pairs. Note that this list of candidate pairs must be added as part of the input to relate the entries of the affinity matrix to the feature points. The indicator variable *x* size varies also accordingly to the symmetric affinity matrix size. Its length corresponds to the column, of line, size of the matrix, and may vary from $N_{P}\times N_{Q}$ to $K\times N_{P}$ depending on the application.

Here, the matching score is completely retained as pairwise geometric only. The individual affinity $M(e_{1},e_{1})$ that represents first order affinity, is set to zero since there is no information about individual affinity. That is to say, all the diagonal values of the affinity matrix are zeros. The pairwise affinity $M\left(e_{1},e_{2}\right)=M\left(ia,jb\right)$ between edges is given by:

(28) $$M\left(ia,jb\right)=max\left(50-d_{ia;jb},0\right),$$

where $d_{ia;jb}$ is the mutual projection error function used in [58] between two candidate assignments (i,a) and (j,b), that includes euclidean distance evaluation $d_{ij}$ between locations of features i,j. The Table 1 summarizes notations and definitions used in this paper.

## 6. Experimental Evaluation

### 6.1. First-Order GM Experiment

The experiments were performed on a CPU Intel(R) Core(TM) i5-4590 3.3 GHz. In this section, we evaluate the proposed feature point matching method by using the Visual Geometry Group dataset (https://www.robots.ox.ac.uk/~vgg/data/). The following will be divided into two parts for experimental description: extract feature points and perform feature matching. For quantitative evaluation, a set of experiments was performed. The proposed algorithm based on Laplace filter and Marr wavelets under entropy method (L_Marr_E) was compared with other classic conventional methods, corner detector [9], Gilles [7], Harris [6], LoG [8] and SIFT [11] algorithms.

#### 6.1.1. Feature Points Extraction

**Figure 8:**
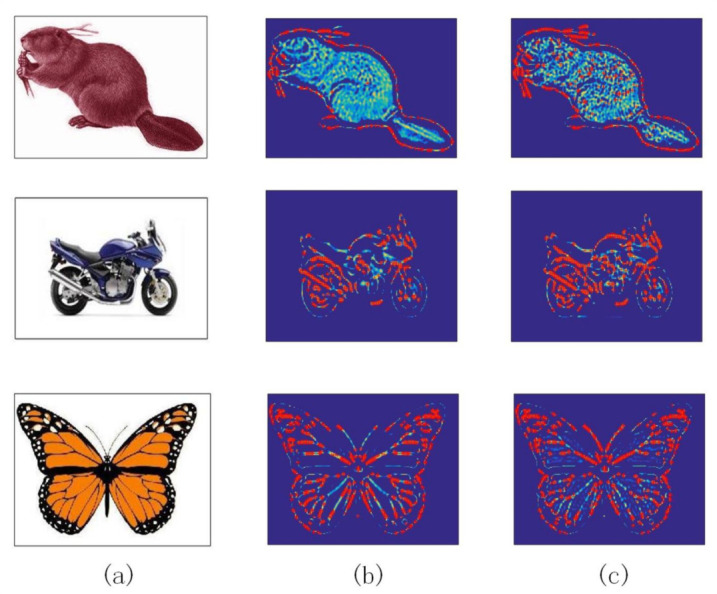
Quantitative experimental analysis of feature point extraction: (**a**) original image, (**b**) feature point extraction without Laplace filter, (**c**) feature point extraction with Laplace filter.

**Figure 9:**
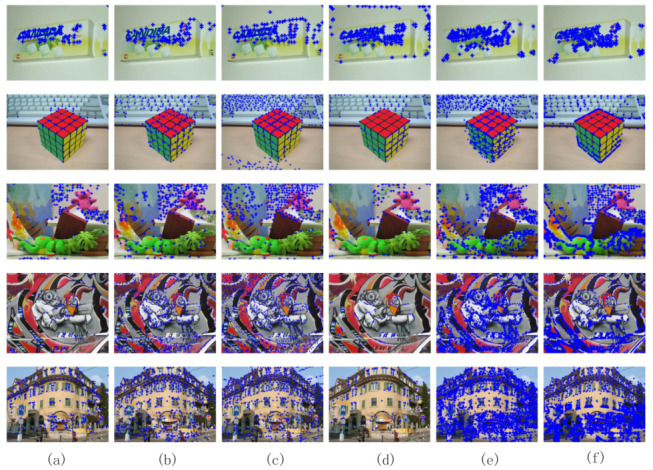
Feature points extraction under different algorithms: (**a**) corner detector (CD) (**b**) Gilles (**c**) Harris (**d**) LoG (**e**) SIFT (**f**) L_Marr_E.

Based on the above experimental theory, the following experimental verification was performed. First, we needed to verify the importance of the Laplacian filtering algorithm at the feature point extraction stage. Figure 8b,c show comparison graphs before and after adding a Laplacian filter. We can clearly see that using Laplace filters could greatly increase the number of feature points detected. Figure 9 shows the feature point detection results between different algorithms: CD, Gilles, Harris, LoG, SIFT, and L_Marr_E. Correspondingly, Table 2 details the specific number of feature points extracted of Figure 9. Compared with the conventional method, this method could extract more feature points.

#### 6.1.2. Feature Points Matching

**Figure 10:**
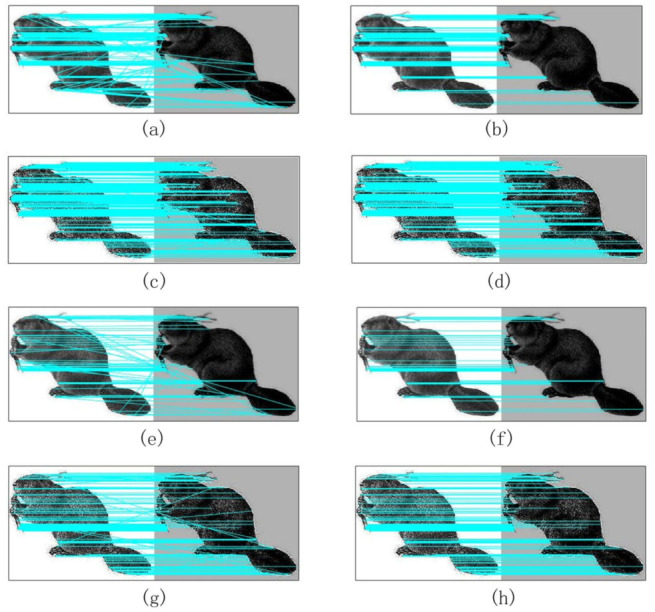
(**a**,**b**) show graph matching without Laplace filter before and after RANSAC optimization; (**c**,**d**) show graph matching under Laplace filter before and after RANSAC optimization; (**e**,**f**) show graph matching using entropy algorithm without Laplace filter before and after RANSAC optimization; (**g**,**h**) show graph matching using entropy algorithm under Laplace filter before and after RANSAC optimization.

The following experiments verified the application of entropy-based refinement in feature matching. Combined with the fragile feature selection stage, ablation analysis provided some valuable intuitions for the pipeline in the feature matching stage. Then, the Figure 10 shows the comparison results, and we found that the RANSAC algorithm could thoroughly eliminate the mismatch points. Besides, the NCC algorithm based on Laplacian filter and Marr wavelet (L_Marr) not only had higher matching accuracy but also could increase the number of correct feature matches than the method without Laplace filter (Marr).

Then we conducted another ablation study. We calculated the five groups of databases as used in Figure 9 and took their final average as shown in Table 3. It summarizes the number of image matching pairs, the number of recall, and time. The computation of graph matching Recall [59] can be defined as follows:

(29) $$Recall=N_{rm}/N_{tm}$$

where $N_{rm}$ is defined as the number of detected true matches after RANSAC removes the mismatched points, and $N_{tm}$ means the total number of correspondences. From the Table 3, we can find that the use of Laplace increases the recall rate, but at the same time it increases the computing time; however, the use of entropy can greatly improve operating efficiency under the premise of basically ensuring the recall value. Therefore, the two complement each other, the combination of Laplace and entropy can better realize the superiority of the proposed algorithm. As shown in the Table 3, the accuracy of the global L_Marr_E method remains very high, even if slightly lower than the accuracy of L_Marr method with no entropy, whereas a substantial computation time acceleration by a factor 5 is a benefit of the method with entropy element.

**Figure 11:**
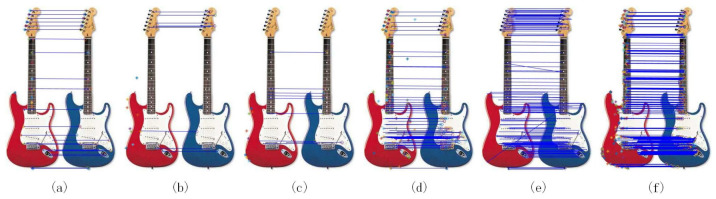
Feature points matching under different algorithms: (**a**) CD (**b**) Gilles (**c**) Harris (**d**) LoG (**e**) SIFT and (**f**) L_Marr_E.

We now selected the L_Marr_E method, which provided the best compromise in both accuracy and computation time in previous tests, as a competitor against other feature point selection methods from literature. We conducted another experiment based on test data from the University of Oxford’s Object Category dataset, which contained 13 different image sets. Among them, seven data sets were selected for comparative analysis in this section. Table 4 shows the average of all image pairs selected from the dataset. The best recall results were obtained through L_Marr_E, which produced the most significant feature points in the two query images. It should be noted that when compared to standard feature point extraction methods, our method provided the best accuracy despite a slight increase of computation time. While entropy accelerated feature point selection in our framework with Laplacian and Marr wavelet, as shown in Table 3, it allowed on the contrary to improve accuracy against other independent standard feature point selection methods, as shown in Table 4. Even when compared to the famous SIFT method, our method remained competitive according to the trade-off between time and accuracy, accuracy being improved at a slight expense of computation time. Note that L_Marr_E also provided the highest number of feature points. Figure 11 shows the corresponding links between images under different algorithms: CD, Gilles, Harris, LoG, SIFT, and L_Marr_E. Therefore, the proposed algorithm ould obtain the better matching effect.

### 6.2. Second-Order GM Experiment

In this subsection, experiments were conducted on a CPU Intel(R) Core(TM) i5-4590 3.3 GHz. We performed experiments on the CMU house image database and real image database. Accuracy, objective score and time were the most important main parameters in the field of graph matching. We needed to use them as criterion when comparing with other different algorithms. The datasets used in this paper, such as CALTECH and CMU house datasets, are the most popular databases for some state-of-the-art algorithms. Although there are still many different databases, in order to facilitate the comparative study of researchers who study graph matching, this article used these most popular databases for easier comparing research. The proposed NCC algorithm was compared with two state-of-the-art methods such as RRWM [18] and SM [29]. All of these different algorithms shared the same images, feature points, and affinity matrices as input data. Each test set had a ground-truth solution for accuracy evaluation.

#### 6.2.1. Presentation

Performance evaluation could be done by computing the affinity score, but more importantly, by evaluating accuracy according to a ground-truth set of true assignment pairs, that reflected the application requirement of graph matching. We also evaluated the computation time. Based on the above IQP, the objective score could be obtained by formula (4). Accuracy could be obtained by dividing the actual correct number of matches detected by the maximum number of ground truth pairs that could be returned, and the formula was as follows:

(30) $$Accuracy=x^{*}*V_{GTbool}/maxGT,$$

where $x^{*}$ is the binary vector solution returned by the algorithm, $V_{GTbool}\in {\left\{0,1\right\}}^{N_{P}N_{Q}}$ is a binary vector representing the ground-truth pairs, and maxGT is the maximum number of true assignments that could be returned when considering a many-to-one mapping, relaxing constraint formula (2). However, solutions returned by the algorithms had to necessarily verify a one-to-one mapping in this section. They verified both constraints formulas (2) and (3).

#### 6.2.2. CMU House Image Matching

**Figure 12:**
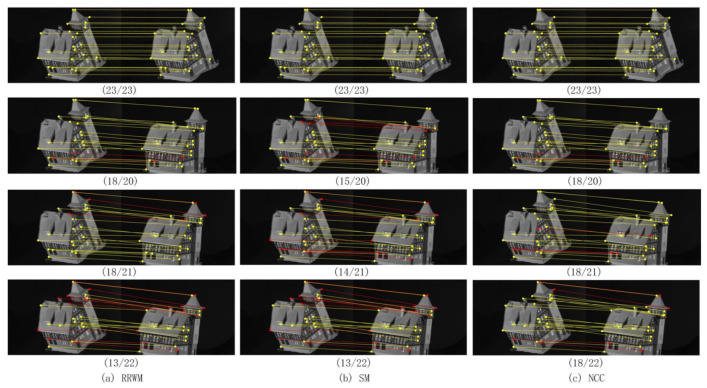
CMU house dataset matching results among (**a**) RRWM, (**b**) SM, and (**c**) NCC algorithms.

Experiments using the CMU house internal sequence dataset (http://vasc.ri.cmu.edu/idb/html/motion/) could evaluate sequence matching of the same object. A total of 110 pictures in this dataset were divided into different sequence gaps (from 10 to 100 with an interval of 10). Therefore, we ended up with ten sets of data pairs. Each pair of image sets consisted of an initial fixed-position picture (sequence 1) and its changing transformations. To evaluate the matching accuracy, 30 landmark feature extraction points were manually tracked and labeled as ground truth on all frames. In this typical classic CMU test, the proposed NCC method was competitive to the RRWM algorithm. The experimental results are shown in Figure 12, and the detailed information of the quantitative evaluation is recorded in Table 5. From Table 5 we can find that in this typical single object with only the angle conversion test, the proposed NCC algorithm was comparable to RRWM in terms of accuracy and score. NCC also cost less computing time than RRWM.

#### 6.2.3. Real Image Matching

**Figure 13:**
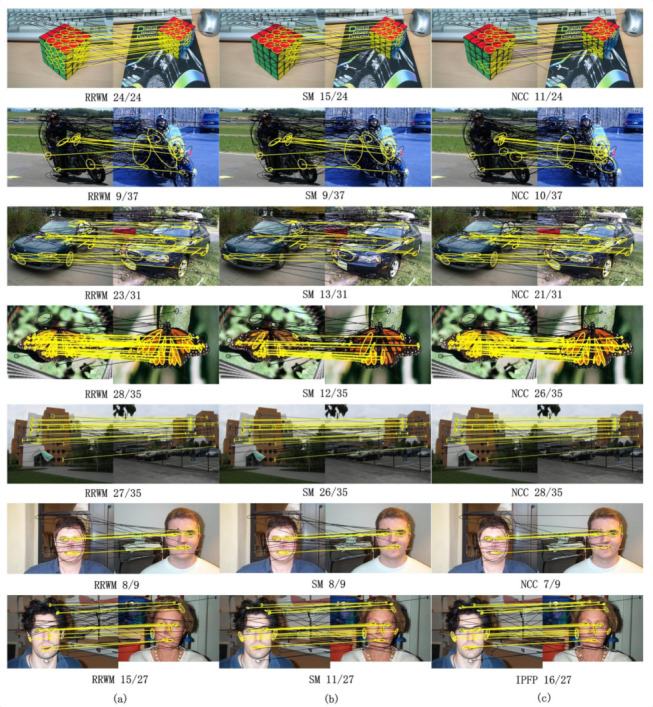
From the left to the right: (**a**) RRWM algorithm, (**b**) SM algorithm, and (**c**) NCC algorithm for graph matching. (The yellow lines represent the correct matching pairs, and the black lines represent the wrong matches.)

In the experiments of real image matching, we used the CALTECH database (https://cv.snu.ac.kr/research/~RRWM/) customized by Cho et al. [18]. In this CALTECH database, it contained 30 different image pairs. All ground truths of these corresponding candidates were manually pre-labeled. Accuracy and objective scores were the main criteria for matching. Table 6 shows the average results of 30 pairs of images obtained by RRWM, SM, and NCC algorithms. As can be seen from the table, the performance of the NCC algorithm was not better than the RRWM and SM algorithms. Figure 13 shows the visualization of feature point connections. Correct and incorrect matches were marked with yellow and black lines, respectively.

Since the second-order graph matching based on IQP was an NP-hard problem, various approximate solutions were used to attempt to solve the pairwise similarity corresponding mapping problem. Leordeanu and Hebert provided a spectral matching (SM) algorithm [29] based on the main strength cluster of the adjacency matrix by finding its principal eigenvector. Reweighted random walks for graph matching (RRWM) algorithm was introduced by Cho et al. [18], and it combined mapping constraints with re-weighted jumping schemes. Both of these two methods implement an iteration loop to compute a principal eigenvector. This basic iteration loop technique belongs to the power iteration method. Although this method cannot guarantee to achieve the final global optimal solution during the operation, it can converge to the fixed point of the tensor. However, NCC algorithm is a single-threaded algorithm but not an iterative method. When the normalized cross-correlation algorithm calculation is over, the entire matching process is completed. It cannot run the optimization iterative until the solution *x* converges. Therefore, the NCC algorithm cannot achieve the expected optimization effect in the second-order matching.

## 7. Conclusions

We study different declinations of feature correspondence problems by the use of the Matlab platform, in order to reuse and provide state-of-the-art solution methods, as well as experimental protocols and input data necessary with evaluation and comparison tools against existing sequential algorithms, most of the time developed in Matlab framework. While feature extraction methods are numerous, it is not straightforward that each could represent the objects to match from a query to a reference image adequately. In order to vary the feature set size while preserving a reasonable recall rate in graph matching, we have proposed a new combination of filters with an entropy-response based selection method. Laplace filter enhances the edges and details of an image. Secondly, Marr wavelets embedded in scale-interaction are used to detect feature points. Then, we use the entropy and brightness response to extract typical feature points. Most importantly, the entropy-based selection method greatly reduces the calculation time. Image matching is achieved by nearest neighbor search using a normalized cross-correlation similarity measure. Finally, the RANSAC process deletes the outlier correspondence to achieve matching optimization. The first-order comparison results show that our algorithm has a higher matching rate for reasonable computation time, despite the augmentation of the number of feature points under the Laplace algorithm. The second-order graph matching is also realized based on the NCC algorithm, which allows us to address graph matching or derived sub-problems with a closed relationship with experiments on IQP models in the Matlab platform. We found that the second-order NCC approach performs competitively to IQP models on CMU images on accuracy and score. The performance looks less satisfactory for real case images of the CALTECH database. In future work, we will improve implementation by exploiting natural parallelism of the method on the GPU platform.

## Figures and Tables

**Table 1:** Summarization of notations.

Notation	Purpose
$G_{P}$	Reference graph
$G_{Q}$	Query graph
*P*	Set of features in $G_{P}$
*Q*	Set of features in $G_{Q}$
$N_{P}$	Total number of data featrues of $G_{P}$
$N_{Q}$	Total number of data featrues of $G_{Q}$
*C*	Mapping constrains
*L*	A set of candidate assignments
i,j	Feature points in $G_{P}$
a,b	Feature points in $G_{Q}$
$e_{1}=\left(i,a\right),e_{2}=\left(j,b\right)$	Candidate assignments
*M*	Affinity matrix
$M(e_{1},e_{2})$	Pairwise affinity
$M(e_{1},e_{1})$	Individual affinity
*S*	Graph matching score
*x*	Indicator vector
$x^{*}$	Optimal solution
$d_{ij}$	Euclidean distances between the point *i* and *j*

**Table 2:** Feature point extraction results under different kinds of algorithms.

Method	CD	Gilles	Harris	LOG	SIFT	L_Marr_E
img.1	72	73	104	174	226	264
img.2	70	246	457	165	980	654
img.3	144	274	341	110	763	1576
img.4	203	844	1133	153	3533	8402
img.5	320	627	696	140	3366	7806

**Table 3:** Experimental comparison results for quantitative analysis of feature point matching.

Methods	$N_{\mathrm{rm}}$	$N_{\mathrm{tm}}$	Recall (%)	Time (s)
Marr	3323	4711	70.58	18.76
L_Marr	10407	10954	95.01	98.02
Marr_E	998	1455	68.59	10.33
L_Marr_E	3523	3740	94.20	21.45

**Table 4:** Feature point matching results under different kinds of algorithms.

	Detector	1st Image	2nd Image	Time (s)	Recall (%)
1	CD	51	53	1.76	0.36
2	Gilles	124	134	2.24	0.16
3	Harris	208	226	2.8	0.35
4	LoG	300	300	4.27	0.27
5	SIFT	1275	1258	5.49	0.48
6	L_Marr_E	1998	1929	7.22	0.52

**Table 5:** Comparative evaluation on CMU database for reweighted random walk method (RRWM), spectral matching method (SM), and normalized cross-correlation (NCC).

	Methods	Accuracy	Score	Time (s)
1	RRWM	92.61	99.93	0.52
2	SM	89.21	94.44	0.07
3	NCC	94.88	95.31	0.23

**Table 6:** Comparative evaluation on CALTECH database for RRWM, SM, and NCC.

	Methods	Accuracy	Score	Time (s)
1	RRWM	61.13	99.92	0.16
2	SM	50.22	79.93	0.02
3	NCC	52.17	74.39	0.06
